# Unravelling the hidden power of esterases for biomanufacturing of short-chain esters

**DOI:** 10.1038/s41598-023-37542-x

**Published:** 2023-07-04

**Authors:** Aditya P. Sarnaik, Somnath Shinde, Apurv Mhatre, Abigail Jansen, Amit Kumar Jha, Haley McKeown, Ryan Davis, Arul M. Varman

**Affiliations:** 1 Chemical Engineering Program, School for Engineering of Matter, Transport and Energy, Arizona State University, Tempe, AZ USA; 2 Bioresource and Environmental Security, Sandia National Laboratories, Livermore, CA USA

**Keywords:** Biotechnology, Chemical biology, Systems biology

## Abstract

Microbial production of esters has recently garnered wide attention, but the current production metrics are low. Evidently, the ester precursors (organic acids and alcohols) can be accumulated at higher titers by microbes like *Escherichia coli*. Hence, we hypothesized that their ‘direct esterification’ using esterases will be efficient. We engineered esterases from various microorganisms into *E. coli*, along with overexpression of ethanol and lactate pathway genes. High cell density fermentation exhibited the strains possessing esterase-A (SSL76) and carbohydrate esterase (SSL74) as the potent candidates. Fed-batch fermentation at pH 7 resulted in 80 mg/L of ethyl acetate and 10 mg/L of ethyl lactate accumulation by SSL76. At pH 6, the total ester titer improved by 2.5-fold, with SSL76 producing 225 mg/L of ethyl acetate, and 18.2 mg/L of ethyl lactate, the highest reported titer in *E. coli*. To our knowledge, this is the first successful demonstration of short-chain ester production by engineering ‘esterases’ in *E. coli*.

## Introduction

With increasing global efforts towards sustainability, esters, being biodegradable, are gaining significant recognition for an array of industrial applications, with a major market share in the cosmetics industry, followed by food, lubricants, pharmaceutical, and coating industries^1,2^. The US esters market, which was 3.8 billion USD in 2019, is estimated to reach 5 billion USD by 2025, primarily driven by increasing demand for emulsifiers and stabilizers in personal care and detergent sectors^3^. Predominantly, short-chain esters such as ethyl lactate and ethyl acetate occupy the commercial market owing to their biodegradable and environment-friendly characteristics^1^.

Esters are traditionally synthesized from carboxylic acids and alcohols in the presence of an acid catalyst, typically concentrated sulfuric acid^4^. However, due to the use of excess alcohol and corrosive chemicals, advanced eco-friendly approaches, like microbial production route, are required to avoid further environmental damage. Microbial cell factories offer a sustainable platform for producing industrial solvents from renewable resources^5–9^. Biocatalytic esterification can provide an eco-friendly alternative, as it occurs at ambient temperature and does not involve any corrosive waste products. Hence, biomanufacturing of green solvents, like esters, could be an effective substitute^10^.

Although microbes have long been exploited as cell factories for the production of various chemicals, the catalytic activity of the enzyme/s involved with the engineered pathway/s remains as one of the major bottlenecks. Hence, mining for new enzymes with better activity becomes essential. Under the current investigation, rigorous bioprospecting was performed to find potent esterifying enzymes from various natural producers^1,11,12^, and *Escherichia coli* was engineered for the ester production.

**Figure 1:**
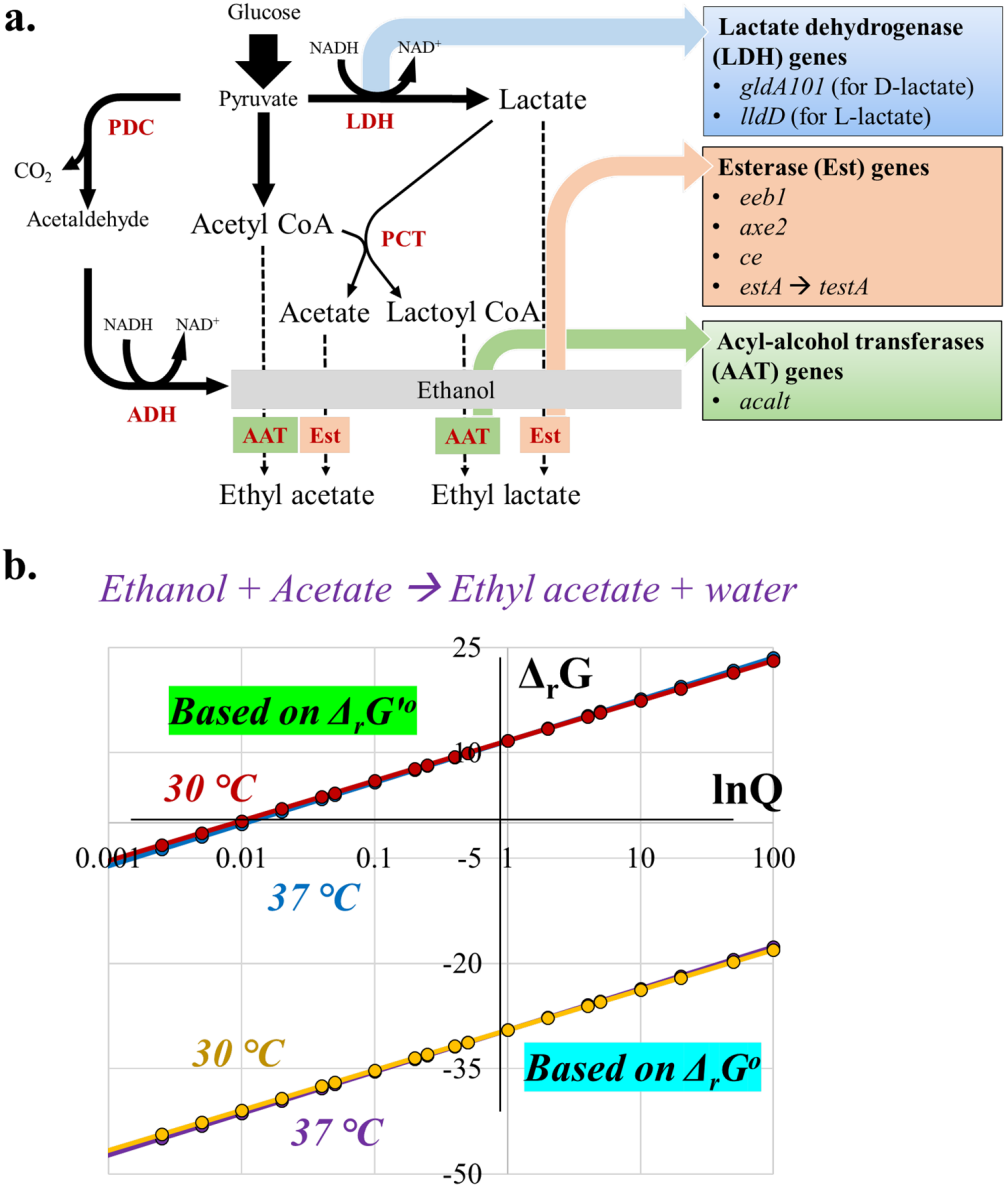
Schematic representation of the metabolic pathways for ester production and in silico Δ_r_G predicted for ester synthesis via esterification: (**a**) Ester bioproduction can be achieved via acyltransferase (using acyl-CoA and alcohol as precursors) or esterase (using organic acid and alcohol as precursors) catalysis. The metabolic pathway for engineering *E. coli* cells for the biosynthesis of short-chain esters (ethyl lactate and ethyl acetate) and precursors thereof, including ethanol, D-lactate, and L-lactate. The thickness of the arrows depicts the average flux in pathway^63^, dotted arrows indicate the esterification reaction through acyltransferase (AAT) or esterase (Est) routes. Heterologous enzymes (LDH, lactate dehydrogenase; ADH, alcohol dehydrogenase; PCT, propionyl CoA synthase; PDC, pyruvate decarboxylase) that have been overexpressed are represented in red color and bold, whereas the genes encoding for AAT, Est and lactate dehydrogenase have been enlisted in a green, orange, and blue box respectively next to the pathway. (**b**) Gibbs free energy (Δ_r_G and Δ_r_G′) estimation for varying reaction quotients (Q) at different temperatures (30 °C and 37 °C) for the production of ethyl acetate via an esterification reaction. Δ_r_G°, standard Gibbs free energy of a reaction; and Δ_r_G′°, standard Gibbs free energy of a reaction at pH 7 and ionic strength of 0.1 M. The image was created using Microsoft PowerPoint and Microsoft Excel.

Naturally, ester biosynthesis can be accomplished through either of the four categories of enzymes: acyltransferases; esterases; hemiacetal dehydrogenases; and monoxygenases^13^. All of the earlier reports on microbial ester production have predominantly overexpressed ‘acyltransferase’ enzymes such as alcohol O-acetyltransferase 1 (Atf1) from yeasts, vesca alcohol acyltransferase (VAAT) from the wild strawberry plant (Fig. 1a)^1,2,14,15^. They require acyl-CoA and alcohol as their substrates, wherein relatively less substrate (acyl-CoA) availability could be one of the primary limitations in acyltransferase catalysis. On the other hand, substrates for ‘esterase’, organic acids and alcohols, are usually accumulated by the microbial hosts like *E. coli* at relatively higher titers (Fig. 1a)^16^. Hence, their direct esterification will be favorable. In addition, a further increase in the precursor titers through metabolic engineering can improve the carbon flux towards the ester production^17,18^. Despite the above advantages, esterases have never been exploited as candidates for ester production via metabolic engineering. A positive Gibbs free energy for biological esterification has been attributed to this limited interest^1,2^. However, there are pieces of evidence for biological esterification by esterases, which contradict these estimations^19–21^. The Gibbs free energy of reaction estimated in these earlier reports were based on an online interface, eQuilibrator^1,2,13^. The eQuilibrator uses a default pH and ionic strength of 7.0 and 0.1 M, respectively, to obtain Gibbs free energy at biological conditions (Δ_r_G′). However, cytoplasmic pH and ionic strength for microorganisms vary widely from this default value^22–24^, and this can introduce error in the Gibbs free energy prediction^25^. In addition, as these estimations involve mathematical models, like the ‘group contribution method’ that have an average error of 9–10 kJ/mol, we were not convinced with the positive Δ_r_G′ estimated with this approach^26,27^. With a wide range of precursor concentrations (Q, reaction quotient) in consideration, Δ_r_G estimated from Δ_r_G^o^ (Gibbs free energy of a reaction at standard conditions) for the esterase catalyzed esterification; turned out to be negative, further adding to the conundrum (Fig. 1b). Therefore, experimental validation of the esterase catalyzed esterification route is important owing to the several advantages for rapid progress in ester synthesis. Comprehending this, under the current study, we have engineered *E. coli* by cloning various esterases, and have analyzed their in vivo esterification potential.

The current work encompasses in silico mining of various esterases from *Brettanomyces bruxellensis* AWRI1499, *Saccharomyces cerevisiae*, *Pichia pastoris,* and *Pseudomonas aeruginosa*. The nucleotide sequences for the esterases identified from *B. bruxellensis* have incorrect non-nucleotide/non-amino acid inclusions in the online databases (NCBI Genbank and Uniprot) and hence, preliminary manual curation was performed based on their sequence homologies. Comparative biochemical analysis of all the enzymes unveiled that esterases could serve as potent esterifying biocatalysts. Furthermore, high cell density (HCD) fermentation and optimization of cultivation conditions showed significant production of ethyl lactate and ethyl acetate from the engineered cells at the laboratory as well as bioreactor scales. Therefore, our research is the first successful demonstration of using esterases to produce short-chain esters: ethyl lactate and ethyl acetate.

## Results

### Gene mining and sequence curation

Bioprospecting for esterifying enzymes was performed to obtain their genetic sequences from the following natural producers: *B. bruxellensis* AWRI1499; *S. cerevisiae*; *Pichia pastoris*; and *Pseudomonas aeruginosa*. As a first step, *B. bruxellensis* proteome was analyzed, and two esterases, acetylxylan-esterase (AXE2) and carbohydrate-esterase (CE) were identified for exploring their esterification potential^28,29^. Gene/polypeptide sequences for the selected enzymes from *B. bruxellensis* had incorrect nucleotide/amino acid inclusions in GenBank/ UniProt. Hence, manual curation was performed based on sequence similarities using *blastn*, *blastp,* and *clustal omega* tools (Supplementary Fig. SI-1a). Local alignment (BLAST) and multiple sequence alignment (ClustalO) of the GenBank/UniProt sequences were performed to obtain the most closely related sequences, and the incorrect inclusions were corrected by replacement with the nucleotide/amino acids from the homologous sequence/s (Supplementary Fig. SI-1a). During replacement, the polarity and the hydrophobicity index of the neighboring amino acids were considered. Final nucleotide sequences were subjected to in silico translation (TRANSLATE tool) and analyzed for their folding using the SWISS-MODEL tool. Similarly, model fermentative yeasts *Pichia pastoris* and *S. cerevisiae* were explored for their inherent esterifying enzymes, acyl-coenzymeA: ethanol O-acyltransferase (AcAlT) and ethyl-ester-synthase-1 (EEB1), respectively.

Like yeasts, pathogenic bacteria like *Pseudomonas* are also known to synthesize potent esterases^30,31^. Interestingly, Esterase-A^32^ is a membrane bound esterase from *Pseudomonas* with its inherent signal peptide, and it is completely annotated in protein repositories, including UniProt. Hence, EstA from *Pseudomonas aeruginosa* was also selected for studying its esterification potential^30^ (Fig. 1a). Further, to increase its soluble cytoplasmic fraction, we truncated EstA by removing the signal sequence to produce tEstA^33,34^ (Supplementary Fig. SI-1b). SDS-PAGE results of the soluble fraction showed a more prominent protein band corresponding to tEstA (~ 66 kDa) than EstA indicating successful solubilization (Supplementary Fig. SI-1b). In total, four different esterases (EEB1, EstA, CE, and AXE2), along with an edited tEstA, and a representative acyltransferase (AcAlT) were considered for heterologous cloning into *E. coli*^35,36^ (Fig. 1a).

### In vitro estimation of esterase activity

**Figure 2:**
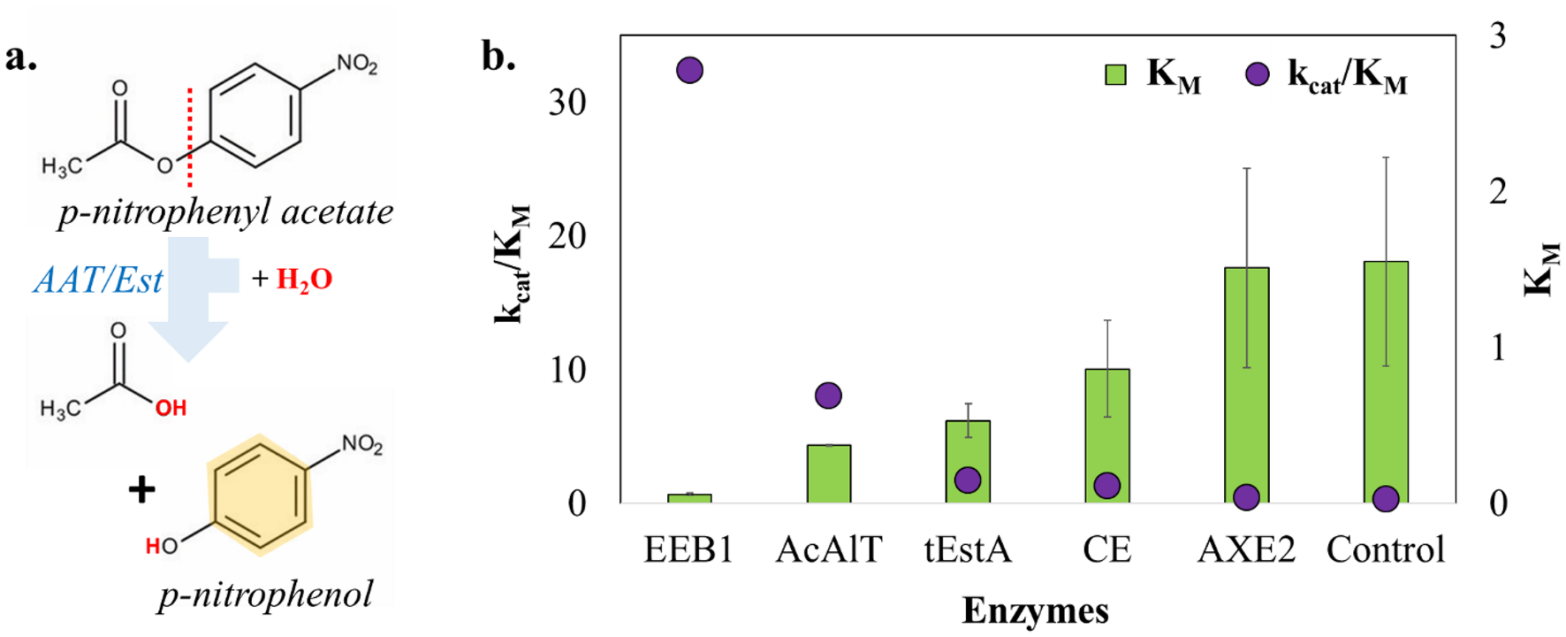
In vitro esterase biocatalysis: (**a**) In vitro esterase assay using artificial substrate p-nitrophenyl acetate (pNPA) encompasses AAT or Est catalyzed substrate hydrolysis to p-nitrophenol (pNP, yellow colored solution), which can be spectrophotometrically quantified. (**b**) Comparative pNPA hydrolysis assay using different crude *E. coli* lysates possessing AAT (AcAlT), esterases (EEB1, tEstA, CE, AXE2), and control (with pET15b)**.** Results indicated a mutually reverse trend of K_M_ and reaction specificity (k_cat_/K_M_). The image was created using Microsoft PowerPoint and Microsoft Excel.

All the genes coding for esterases and an acyltransferase were codon optimized, synthesized, and successfully cloned under the control of T_7_ promoter (*P*_*T7*_). Phase I *E. coli* strains were constructed by transforming *E. coli* BL21-DE3 with the above plasmid constructs (Supplementary Fig. SI-2). The whole cell lysates of the induced Phase I strains were analyzed for ester hydrolysis activity based on pNPA colorimetric assay. *k*_*cat*_/K_M_ (catalytic efficiency) and K_M_ values revealed that EEB1 exhibited the highest catalytic efficiency for pNPA hydrolysis over other candidates (Fig. 2). AcAlT, despite being acyltransferase, showed the second highest efficiency and relatively lower K_M_ (higher affinity for the substrate), presumably due to its broader substrate specificity (Fig. 2). CE and tEstA, on the other hand, indicated relatively moderate hydrolysis activity with higher K_M_ values than that of AcAlT (Fig. 2). However, AXE2 exhibited lower catalytic efficiency, but higher K_M_ comparable that of the BL21-pET lysate control. Therefore, all the enzymes were further investigated for their in vivo esterification potential.

### Metabolic engineering of *E. coli* for in vivo esterification

As a next step to assess the esterification potential of these heterologous enzymes, the Phase I strains were investigated for the production of ethyl lactate. This part of the study leveraged on the inherent ability of *E. coli* to produce both ethanol and lactate. BL21-pET strain possessing empty plasmid was used as a negative control. Therefore, first, the control strain BL21-pET possessing empty plasmid (Table 1), was explored for the production of lactate and ethanol through high-cell density (OD_600_ = 3.0, HCD3) fermentation. Results indicated that the strain could synthesize lactate (3.8 g/L) and ethanol (0.7 g/L) as expected (Supplementary Fig. SI-3a). However, as the ethanol concentration was relatively low, preliminary screening of all the Phase I strains was performed with HCD3, with an external doping of 10 g/L ethanol. However, the ethyl lactate titers amongst the Phase I strains were very less (< 0.1 mg/L) indicating that sole external supplementation of the precursors may not be sufficient (Supplementary Fig. SI-3b). Therefore, the Phase I strains were engineered with the pathways for overproduction of ethanol and lactate, forming Phase II strains (Supplementary Fig. SI-2). The Phase II strains had overexpression of the genes for stereospecific lactate (*gldA101* for D-lactate or *lldD* for L-lactate), and ethanol (*pdc* and *adh* genes from *Zymomonas mobilis*) production (Table 1).

**Table 1:** Strains and plasmids used in this study.

Strain	Plasmid	Gene (Enzyme)	Source organism	Lactate dehydrogenase gene	*pct* gene	pACYDuet-*pdc*-Zm-*adhB*-Zm
Phase I strains						
SSL26	pAPS07	*acalt* (Acyl-coenzymeA: ethanol O-acyltransferase)	*Komagataella phaffii ATCC 20,864* (*Pichia pastoris*)	*–*	–	–
SSL31	pAPS08	*eeb1* (Medium chain fatty acid ethyl ester synthase/esterase 1)	*S. cerevisiae*	*–*	–	–
SSL42	pAPS19	*axe2* (Acetyl xylan esterase)	*B. bruxellensis AWRI 1499*	*–*	–	–
SSL43	pAPS20	*ce* (Carbohydrate esterase family 9)		*–*	–	–
SSL52	pAJ01	*estA* (Esterase A)	*Pseudomonas aeruginosa*	*–*	–	–
SSL55	pAJ04	*testA* (truncated Esterase A)		*–*	–	–
Phase II strains						
SSL64	pAPS21	*acalt* (Acyl-coenzymeA: ethanol O-acyltransferase)	*Komagataella phaffii ATCC 20,864* (*Pichia pastoris*)	*glda*	**√**	**√**
SSL65	pAPS23			*lldD*	**√**	**√**
SSL66	pAPS22	*eeb1* (Medium chain fatty acid ethyl ester synthase/esterase 1)	*S. cerevisiae*	*glda*	**√**	**√**
SSL67	pAPS24			*lldD*	**√**	**√**
SSL72	pMN05	*axe2* (Acetyl xylan esterase)	*B. bruxellensis AWRI 1499*	*glda*	**√**	**√**
SSL73	pMN06			*lldD*	**√**	**√**
SSL74	pMN07	*ce* (Carbohydrate esterase family 9)		*glda*	**√**	**√**
SSL75	pMN08			*lldD*	**√**	**√**
SSL76	pAJ02	*estA* (Esterase A)	*Pseudomonas aeruginosa*	*glda*	**√**	**√**
SSL77	pAJ03			*lldD*	**√**	**√**
SSL79	pAJ05	*testA* (truncated Esterase A)		*glda*	**√**	**√**
SSL80	pAJ06			*lldD*	**√**	**√**
Intermittent strains						
SSL36	pAPS21	*acalt* (Acyl-coenzymeA: ethanol O-acyltransferase)	*Komagataella phaffii ATCC 20,864* (*Pichia pastoris*)	*glda*	**√**	–
SSL37	pAPS23			*lldD*	**√**	–
SSL38	pAPS22	*eeb1* (Medium chain fatty acid ethyl ester synthase/esterase 1)	*S. cerevisiae*	*glda*	**√**	–
SSL39	pAPS24			*lldD*	**√**	–
SSL48	pMN05	*axe2* (Acetyl xylan esterase)	*B. bruxellensis AWRI 1499*	*glda*	**√**	–
SSL49	pMN06			*lldD*	**√**	–
SSL50	pMN07	*ce* (Carbohydrate esterase family 9)		*glda*	**√**	–
SSL51	pMN08			*lldD*	**√**	–
SSL53	pAJ02	*estA* (Esterase A)	*Pseudomonas aeruginosa*	*glda*	**√**	–
SSL54	pAJ03			*lldD*	**√**	–
SSL56	pAJ05	*testA* (truncated Esterase A)		*glda*	**√**	–
SSL57	pAJ06			*lldD*	**√**	–
BL21-pET	pET15b	None	None	*–*	**–**	–

**Figure 3:**
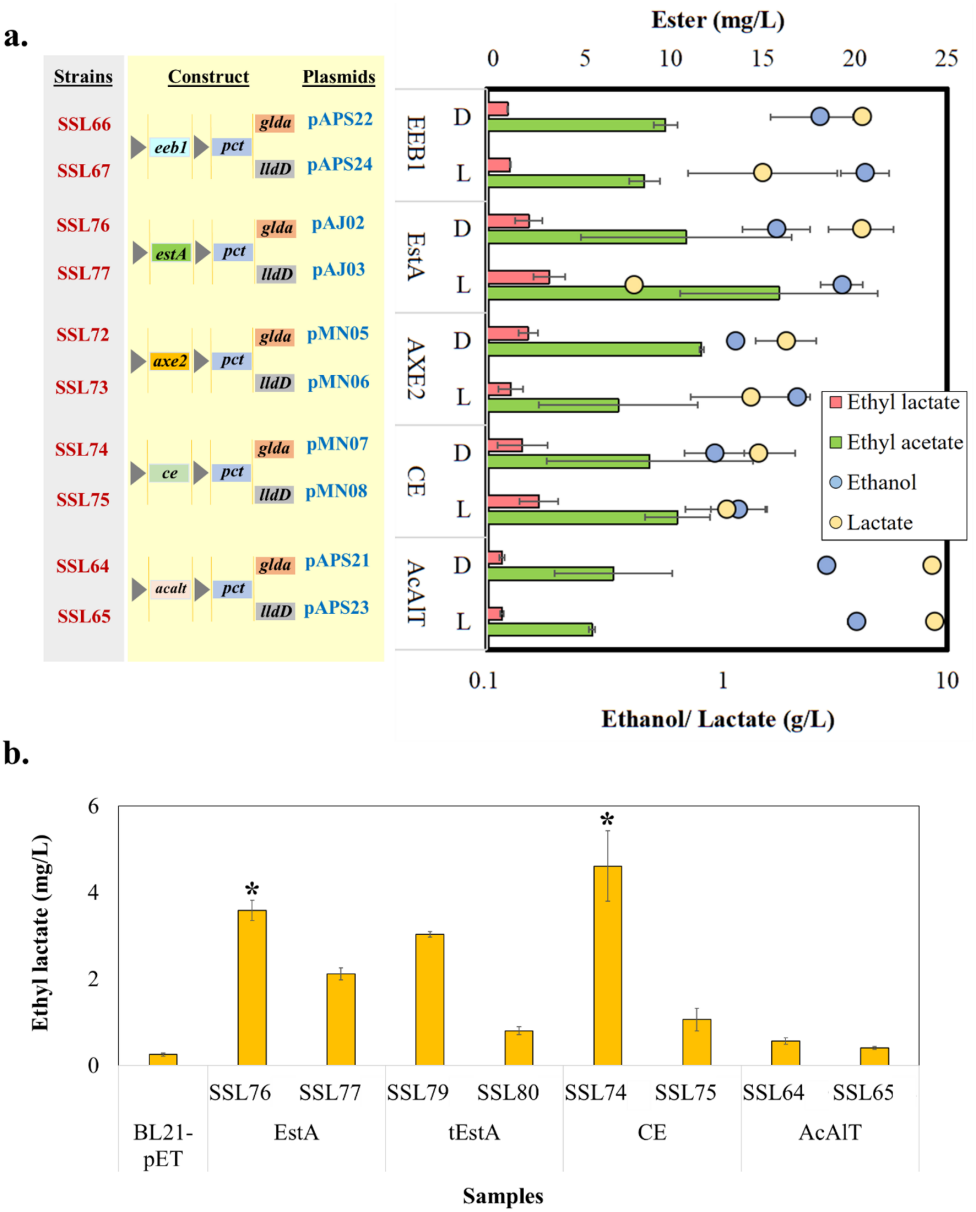
Ester production using high cell density fermentation: (**a**) Preliminary analysis using high cell density HCD3 has been represented as a comparative plot with concentrations of esters and precursors thereof. Corresponding constructs with D- or L-lactate dehydrogenases have been enlisted. The experiment was performed with intermittent aliquot sampling. (**b**) Ethyl lactate production was analyzed at HCD10 with sample-wise cultivation method, where every sample variant and the replicate was cultivated in a separate tube to avoid any oxygen interference during sampling. *E. coli* strains possessing esterases EstA, tEstA and CE, along with AcAlT and control (BL21-pET with empty plasmid) showed higher ethyl lactate production with D-lactate isomer than L-lactate, indicating superior enantio-selectivity of the enzymes for D-lactic acid. AcAlT possessing strains exhibited the least ethyl lactate production over those possessing the esterases. The image was created using Microsoft PowerPoint and Microsoft Excel.

Fermentation studies were conducted with the Phase II strains, to screen for ester production. The preliminary fermentation (OD_600_ = 3.0, HCD3) results indicated that Phase II strains produced significantly higher ester titers than Phase I strains, which could be attributed to the cloned ethanol and lactate over-production pathways (Fig. 3, Supplementary Fig. SI-3b). It was also observed that ethyl acetate was synthesized at higher titers across all the strains in comparison to ethyl lactate. For instance, SSL77 gave the highest ester titers with 15.8 mg/L of ethyl acetate and 3.4 mg/L of ethyl lactate. In addition, the strains expressing esterases EEB1 (SSL66, SSL67), AXE2 (SSL72, SSL73), CE (SSL74, SSL75), and EstA (SSL76, SSL77), exhibited higher ester titers in comparison to the strain expressing the acyltransferase AcAlT (SSL64, SSL65) (Supplementary Fig. SI-4a; Fig. 3a).

Based on the ester titers from the preliminary screening and considering the possible interference of atmospheric oxygen in fermentation during intermittent aliquot sampling, the Phase II strains were subjected to higher cell density fermentation (OD_600_ = 10.0, HCD10) using a sample-wise cultivation strategy. Unlike aliquot sampling, where a small portion of the sample was removed for analysis from the same culture tube every time, sample-wise cultivation strategy employed a separate set of tubes for every sample variant and the replicate. The strategy was implemented with (Supplementary Fig. SI-4b) and without external supplementation of ethanol, and corresponding D- or L-lactic acid. Strains possessing *gldA101* gene (SSL64, SSL74, SSL76, SSL79) produced more ethyl lactate than their counterparts possessing *lldD* gene (SSL65, SSL75, SSL77, SSL80) (Fig. 3b). The highest ethyl lactate titers were exhibited with doping by SSL74 containing CE (8.2 ± 0.4 mg/L) and SSL76 containing EstA (9.5 ± 0.4 mg/L) (Fig. 3b). A quick study conducted by analyzing the spent medium showed that despite increase in the lactate production by the Phase II strains (0.35 g/L in WT to 1.45 g/L in SSL74), there was no effect on the acetate (1.5 g/L in WT and 1.6 g/L in SSL74) produced from acetyl CoA^37^. This observation supported the fact that pyruvate to acetyl CoA flux was unaffected by the overexpression of lactate genes^38,39^. However, well-controlled reactor-scale studies were necessary to comprehend the maximum ester biosynthesis potential of these strains. Therefore, batch fermentation with 60 g/L of glucose in hybrid M9 media was performed in a bioreactor to evaluate the ability of SSL74 and SSL76 to produce the short-chain esters (ethyl lactate and ethyl acetate) under anaerobic conditions.

### Bioreactor study with high cell density fermentation in ‘batch’ mode

**Figure 4:**
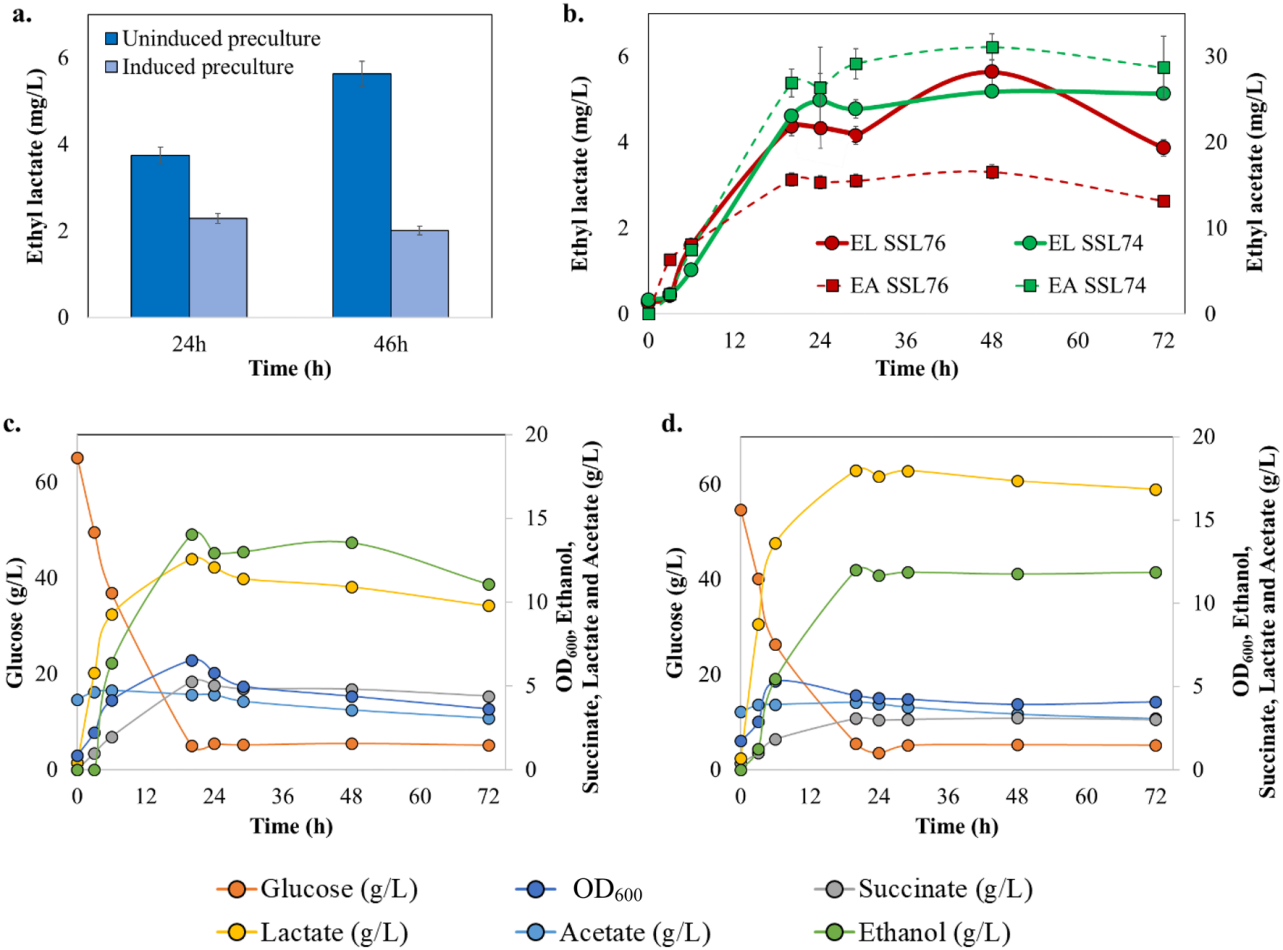
Batch fermentation analysis of engineered *E. coli* strains SSL74 and SSL76 in a 1 L bench-top bioreactor at pH 7 and under anaerobic conditions. (**a**) *E. coli* SSL74 uninduced vs induced pre-culture cultivation. Based on the results, further fermentations were carried out with uninduced pre-cultures. (**b**) *E. coli* SSL74 and *E. coli* SSL76 ethyl lactate and ethyl acetate concentration time curves, (**c**) SSL74 OD_600_, glucose, lactate, acetate, succinate and ethanol concentration time curves, (**d**) SSL76 OD_600_, glucose, lactate, acetate, succinate and ethanol concentration time curves. The image was created using Microsoft PowerPoint and Microsoft Excel.

Although all the earlier experiments were performed by inducing the seed or pre-culture before subjecting it to the high cell density fermentation, to the best of our knowledge, there was no literature available on the effect of pre-culture induction on fermentation titers. Therefore, we studied this effect at the bioreactor scale with SSL76 strain. The seed culture was grown aerobically in a 1 L bioreactor, induced with 0.1 mM of IPTG and incubated for 18 h at 30 °C. The induced pre-culture was subjected to high cell density (with initial OD_600_ ~ 4 to 5) in a batch cultivation mode and was compared for ethyl lactate production with un-induced pre-culture fermentation. Batch fermentation with un-induced pre-cultures showed higher titer for short-chain esters in comparison to that with the induced pre-cultures, at all the time points (Fig. 4a). Hence, for further bioreactor fermentations, IPTG induction was used only during the production phase. Separate set of optimization studies was carried out and the optimal IPTG concentration of 0.1 mM was selected for induction under this study (Supplementary Fig. SI-6a). Detailed optimization studies are mentioned in the Supplementary information SI-6.

Uninduced pre-cultures of SSL74 and SSL76 were subjected to high cell density batch fermentation. The kinetics of glucose, biomass, succinate, lactate, acetate, ethanol, and short-chain esters under batch cultivation mode are shown in Fig. 4b–d. Results showed that short-chain esters production peaked at 29 h (ethyl acetate (25.7 mg/L) and ethyl lactate (4.5 mg/L)) and then leveled off at later stage (Fig. 4b). Similar trend was observed for production of precursors indicating the complimentary correlation of ester production with precursors production. At 20 h of *E. coli* SSL74 cultivation, 12.6 g/L of lactic acid was produced under anaerobic conditions along with 14 g/L, 6.5 g/L and 5.2 g/L of ethanol, acetic acid and succinic acid, respectively. After 20 h, production of precursors leveled off (Fig. 4c). Similarly, both precursors and esters productions peaked for SSL76 at 20 h as well (Fig. 4d). Secondly, 95% and 85% glucose was consumed at 20 h while ethyl lactate and ethyl acetate were constantly produced by *E. coli* SSL74 and *E. coli* SSL76 strains, respectively (Fig. 4c,d).

### Bioreactor study with high cell density fermentation in ‘fed-batch’ mode

**Figure 5:**
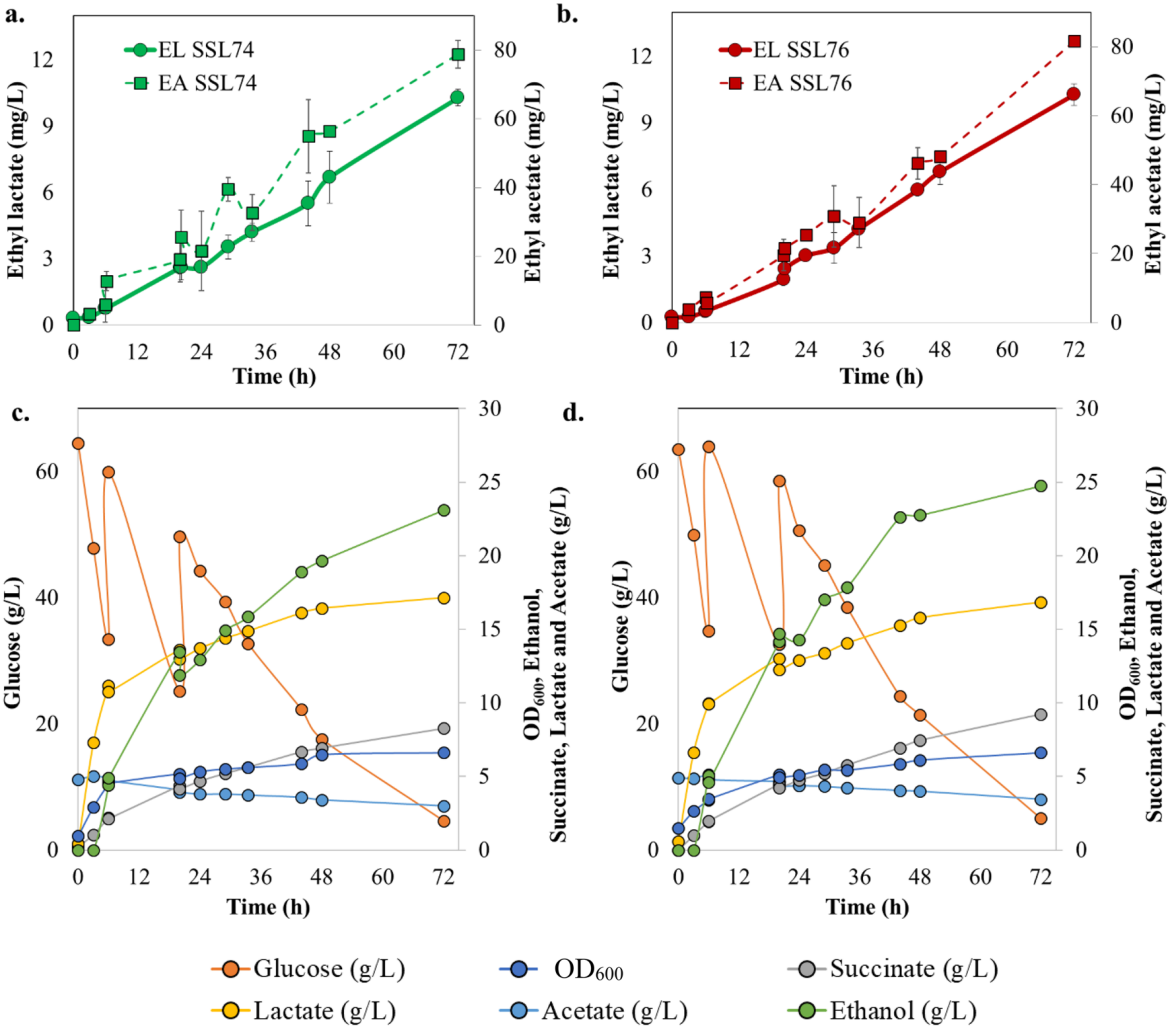
Fed-batch fermentation analysis of recombinant *E. coli* SSL74 and SSL76 strains in a 1 L bench-top bioreactor at pH 7. (**a**) *E. coli* SSL74 ethyl lactate and ethyl acetate concentration time curves, (**b**) *E. coli* SSL76 ethyl lactate and ethyl acetate concentration time curves, (**c**) SSL74 OD_600_, glucose, lactate, acetate, succinate and ethanol concentration time curves, (**d**) SSL76 OD_600_, glucose, lactate, acetate, succinate and ethanol concentration time curves. The image was created using Microsoft PowerPoint and Microsoft Excel.

To maximize the short-chain esters titers, anaerobic dual-pulse fed-batch cultivation was conducted at pH 7 by addition of 25 g/L of glucose at 6 h and 20 h. As seen in Fig. 5, dual-pulse fed-batch cultivation approach resulted in more than three-fold increase in ethyl acetate production (from 25.7 to 80 mg/L) and two-fold increase in ethyl lactate production (from 4.5 to 9 mg/L) in comparison to batch mode cultivation (Fig. 5a,b). In contrast to batch mode, we observed constant increase in ethyl acetate and ethyl lactate production beyond 20 h. Lactic acid, acetic acid, and ethanol productions were also observed to be gradually increasing suggesting that the availability of higher precursors concentration favored the esters production by engineered strains.

**Figure 6:**
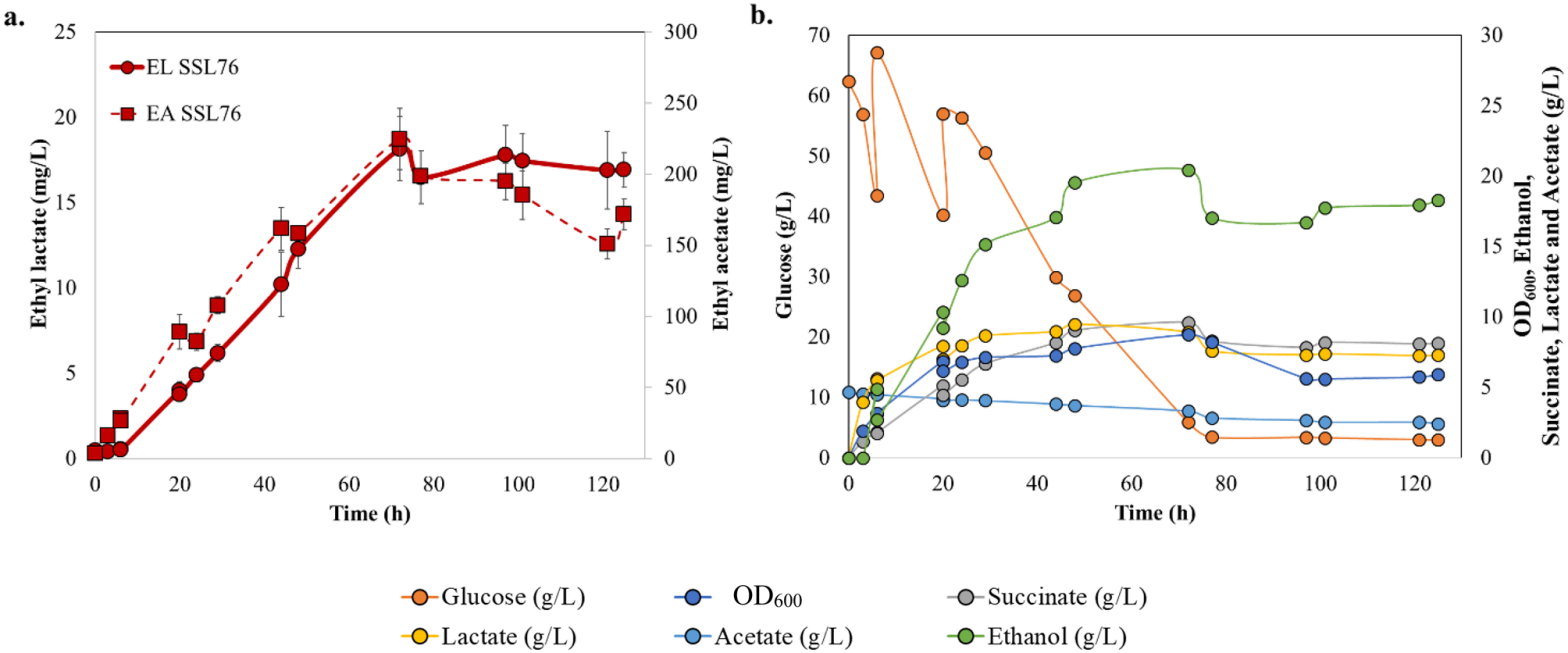
Fed-batch fermentation analysis of recombinant *E. coli* SSL76 strains in a 1 L bench-top bioreactor at pH 6. (**a**) *E. coli* SSL76 ethyl lactate and ethyl acetate concentration time curves at pH 6, (**b**) SSL76 OD_600_, glucose, lactate, acetate, succinate and ethanol concentration time curves at pH 6. Concentrations for pH 6 experiment were corrected to the added volumes of acid and base for pH adjustment. The image was created using Microsoft PowerPoint and Microsoft Excel.

As discussed earlier, pH could be one of the limiting parameters during high cell density cultivation and could significantly affect ester biosynthesis^40,41^. Both the strains, SSL74 and SSL76, were equally efficient in ester production (Fig. 5a,b). Hence, only SSL76 was selected as a representative strain for the further study. To analyze the effect of pH, SSL76 was used as a representative platform strain and fed-batch cultivation was conducted at pH 6. At 72 h, despite relatively lower titers of ethanol, lactate and acetate in the medium than those with pH 7.0 (Fig. 6b), and maintaining the same cell density and glucose feeding, there was two-fold increase in the ethyl lactate production (18.2 mg/L) and three-fold increase in ethyl acetate production (225 mg/L) over pH 7 was observed (Figs. 6a and 5b). This suggested that pH was a crucial factor for short-chain esters production using esterases.

## Discussion

Esters are one of the major aroma components in wine and beer, produced due to the action of yeasts such as *Saccharomyces cerevisiae* and *Brettanomyces bruxellensis*^1,29,42,43^. *B. bruxellensis* is a non-conventional yeast used in the fermentation of craft and specialty beers and natural wine for its ability to produce secondary metabolites like esters during fermentation. It enhances the taste and aroma of the final product^44^. Like yeasts, pathogenic bacteria, e.g. *Pseudomonas* are also known to synthesize potent esterases involved with a rhamnolipid synthesis which exhibits an important role in their swarming motility, pathogenicity, outer membrane, and biofilm formation^30,31^. Hence, as a first step, mining and bioprospecting of various esterifying enzymes was performed. In total, five different esterases (EstA from *P. aeruginosa* along and its truncated variant tEstA, AXE2 and CE from *B. bruxellensis*, EEB1 from *S. cereviciase*), and an acyltransferase, AcAlt from *Pichia pastoris* were selected and considered for heterologous cloning into *E. coli*^35,36^ (Fig. 1a). Phase I strains were constructed by transforming *E. coli* BL21-DE3 with the plasmid constructs possessing only the esterifying enzyme gene under *P*_*T7*_ (Supplementary Fig. SI-2). pNPA assay, performed using the whole cell lysates of their induced biomass, exhibited the following trend of their catalytic efficiencies for ester hydrolysis; AXE2 < CE < tEstA < AcAlT < EEB1. In principle, lower catalytic efficiency for ester hydrolysis, would mean higher esterification potential. So, purportedly AXE2 must be a better esterase. However, AXE2 had a weaker affinity towards the substrate as observed from its K_M_ value. Therefore, we investigated all the enzymes for their in vivo ester production.

As a next step, the Phase I strains were investigated for the production of ethyl lactate. First, the inherent ability of the control strain, BL21-pET to produce ethanol and lactate was analyzed. As BL21-pET produced relatively less ethanol (Supplementary Fig. SI-3a), preliminary screening of all the Phase I strains was performed with an external ethanol supplementation. Such exogenous supplementation could provide better acid:alcohol ratio, thereby driving the equilibrium to ester production^41^. However, they showed very low ethyl lactate titers indicating that sole external supplementation of precursors may not be sufficient. Increasing the intracellular levels of the precursors will be essential for improving the ester synthesis (Supplementary Fig. SI-3b). Hence, two distinct metabolic modules were engineered into *E. coli*: for precursor (alcohol and organic acid) overproduction, and for esterification (Fig. 1a), forming Phase II strains (Supplementary Fig. SI-2, Table 1). The comparative analysis of Phase II strains with intermittent aliquot sampling confirmed the in vivo esterification potential of esterases, giving significantly higher ester titers than the Phase I strains at HCD3 (Fig. 3a). Further analysis of Phase II strains was performed with sample-wise cultivation (HCD10) strategy, to avoid any oxygen interference faced during aliquot sampling. It was done with and without external doping of ethanol, to estimate whether higher precursor concentrations could drive more ester biosynthesis^41^. The results supported this hypothesis (Fig. 3b, Supplementary information SI-4b), indicating that increase in the precursor concentrations could improve the ester titers and it can be very well achieved at the controlled bioreactor scale. To reduce competition between cell growth and metabolite production, the fermentation was carried out in two stages; the first aerobic growth stage where the cells were grown till mid-exponential phase, and the second stage was maintained under anaerobic conditions to increase the production of esters. Results also exhibited SSL74 (possessing CE) and SSL76 (possessing EstA) as the potent candidates for short-chain ester biosynthesis (Fig. 3b). In addition, the esterases were more selective for D-lactate over L-lactate as one of the esterification substrates (Fig. 3b). Multiple parameters; IPTG concentration, phase for induction, pre-culture incubation temperature, and fermentation temperature were optimized (Supplementary information SI-6) for improving the ester production. Ultimately, to analyze the esterification potency of these best performing esterases (CE and EstA) at higher scale and under controlled environmental conditions, scale-up studies were carried out with a bench-top bioreactor in batch mode.

Initially, the effect of pre-culture induction on ester production was analyzed in the bioreactor. To the best of our knowledge, there was no literature available on this effect. For this experiment, the results showed that the uninduced pre-cultures produced higher titers of ethyl lactate with high cell density fermentation than the induced ones (Fig. 4a). The lower titers with induced pre-cultures could be associated with the preliminary metabolic burden, which may have led to decreased productivity^45–49^. Secondly, it is reported that high expression of heterologous proteins results in an adverse and unpredictable outcome on the induced cell development. In contrast, too low protein expression levels result into reduced encounter frequency between the recombinant protein and the precursors^45–48,50^. Hence, the bioreactor studies were carried out under optimized conditions and cultures were induced only during the production phase (Supplementary information SI-6, Fig. 4a).

Despite optimally controlled parameters in batch fermentation, ethyl lactate titers obtained with SSL74 and SSL76 were almost equivalent to the ones obtained in the shake-flasks (Fig. 4b). These titers could be attributed to the substrate limitation and/ or pH changes during the batch mode^51^. Therefore, for further improvement in the ester titers, anaerobic dual-pulse fed-batch cultivation was conducted at pH 7, which resulted in the two–threefold improvement in the ester titers. The results indicated that pH maintenance and glucose supplementation resulted in gradual increase in precursor concentrations, thereby improving the ester production from the engineered strains (Fig. 5a–d). However, pH could be one of the limiting parameters during high cell density cultivation and could significantly affect ester biosynthesis^40,41^. Difference in the pH can lead to variable ionic forms of the enzyme active site, ultimately affecting the enzyme activity^52^. In addition, only undissociated acids can participate in an esterification reaction, and the lower pH leads to higher fraction of such undissociated acid molecules in the system^41^. Hence, optimal esterification can be achieved at lower pH. On the other hand, for *E. coli*, it was reported that the pH values of less than 6.0 would negatively affect the cell growth^52,53^. Therefore, for in vivo esterification, it was necessary to select an optimal pH at which the enzyme activity would be relatively high and at the same time without negatively affecting the cell growth^54^. Hence, a separate set of in vitro study was performed for pH optimization. The results exhibited the highest ester production at pH 6.0 (Supplementary information SI-8). Therefore, to analyze the effect of pH 6.0 on in vivo esterification, SSL76 was used as a representative strain, and dual-pulse fed-batch cultivation was conducted at pH 6. Our results supported the earlier finding, that at pH 6, the ethyl acetate titer improved threefold, whereas the ethyl lactate titer increased by twofold over those observed under pH 7 (Fig. 6a). This suggested that controlled lower pH and dual-pulse of glucose during fed-batch cultivation were crucial factors for increasing the esterase catalyzed short-chain ester production from the engineered *E. coli*.

## Conclusion

In this study, we evaluated various esterases for their esterification potential to produce short-chain esters, ethyl lactate and ethyl acetate. The fermentation parameters were optimized at the flask as well as the bioreactor scales for producing esters at higher titers. Overall, the results revealed that abundant availability of metabolic precursors ethanol, lactic acid, and acetic acid favored the short-chain esters production, while pH played a critical role in maximizing the enzyme activity for product formation. It was evident that EstA (from *P. aeruginosa*), and CE (from *B. bruxellensis*) showed comparable efficiencies in short-chain ester production. Therefore, the fed-batch fermentation by SSL76 strain (possessing EstA) overexpressing the genes for both ethanol and lactate synthesis, resulted in 225 mg/L of ethyl acetate and 18.2 mg/L of ethyl lactate at pH 6. This is the highest reported ethyl lactate titer from an engineered *E. coli*. Further improvement in esterification rate via esterases can be achieved through protein engineering and/or reactive extraction approaches using organic solvents. Conclusively, our study has successfully demonstrated the esterification potential of esterases, and we envision that in the future this strategy can be extended to other established and emerging model microbial hosts.

## Materials and methods

### Reagents and media

All reagents were purchased from Sigma Aldrich (St. Louis, MO), unless otherwise noted. LB and hybrid M9 medium (100 mL/L of 10X M9 salts: 67.8 g/L Na_2_HPO_4_, 30 g/L KH_2_PO_4_, 5 g/L NaCl, 10 g/L NH_4_Cl; 1 mL/L of 1 M MgSO_4_, 1 mL/L of 1 M CaCl_2_, 1 mL/L of 1 g/L Thiamine.HCl, 5 g/L of yeast extract, 1 mL/L of trace metal solution: 0.15 g/L H_3_BO_4_, 0.065 g/L CoSO_4_, 0.05 g/L ZnSO_4_.7H_2_O, 0.015 g/L MnCl_2_.4H_2_O, 0.015 g/L NaMo_4_.2H_2_O, 0.01 g/L NiCl_2_.6H_2_O, 0.005 g/L CuSO_4_.5H_2_O, 3 g/L ferric ammonium citrate) were used for the cultivation^2,55–57^. LB broth and M9 salts were purchased from BD Difco™ (Fisher scientific, AZ). Glucose was added to the final concentrations of 25–100 g/L (2.5–10%) to the medium based on experimental requirement. Working antibiotic concentrations were as follows: carbenicillin (100 µg/mL), chloramphenicol (25 µg/mL).

### Strain construction

All the esterase and acyltransferase genes were codon optimized and synthesized from Twist Biosciences, USA. The mutated glycerol dehydrogenase gene (*gldA101*) encoding for D-lactate hydrogenase activity was amplified from the plasmid by pYY1^58^. L-lactate dehydrogenase gene (*lldD*) was amplified from *E. coli* K12 (MG1655) gDNA. pACYDuet-*Zm-pdc-Zm-adh* plasmid possessing genes for cloning ethanol biosynthesis pathway was a gift from Dr. David Nielsen (Arizona State University, Tempe). Primary plasmid construction and cloning was performed in *E. coli* DH5α. For protein expression and further assays, all the plasmids were transformed in *E. coli* BL21-DE3 strains (Phase I strains) (Supplementary Fig. SI-2). Dual chemical transformation was performed for Phase II strains possessing both the plasmids; one possessing lactate dehydrogenase gene, esterifying enzyme gene and *pct* in pET15b plasmid while the other possessing ethanol biosynthesis pathway genes in pACYDuet plasmid (Supplementary Fig. SI-2). All the expression strains have been enlisted in Table 1 with their corresponding plasmid compositions. Steps involved in plasmid construction have been schematically represented in SI-2. Primers used for gene amplifications and cloning have been enlisted in Supplementary Table SI-7.

## In vitro esterase assay

All the esterases were simultaneously screened^59^ using *p*-nitrophenyl acetate (pNPA) as a substrate for hydrolysis. Phase I strains were grown in LB medium with 100 µg/mL carbenicillin and induced using 1 mM IPTG during mid-exponential phase. The cells were harvested after 18 h of growth at 37 °C at 180 rpm. The pellets were stored at − 80 °C and lysed by in-house designed lysis strategy using SoluLyse^60^. Protein concentration of the cell lysate was measured using Bradford’s method^60^ and was used as a crude enzyme for the esterase assay. 5 mM pNPA was prepared in 1:1 ethanol–water mixture. The assay was performed using reaction concentrations of 70 µg/mL of lysate protein and 0.1 mM of pNPA in a 0.1 M Na-phosphate buffer (pH 7) at 37 °C for 1 h in a microtiter plate at a final volume of 200 µL in triplicates^61,62^. The absorbance was recorded at 410 nm after 30 min and the enzyme activity parameters (K_M_, k_cat_/K_M_) were calculated.

### High cell density cultures

Seed cultures were grown overnight at 37 °C by inoculating the Phase II strain stocks in 5 mL LB medium with antibiotics. 1% (v/v) seed culture was inoculated in 200 mL LB medium in 1 L flask with appropriate antibiotics and incubated in shaker with 250 rpm at 37 °C. Once pre-culture attains the OD_600_ of 0.5–0.6 after 2.5–3 h, cultures were induced with 1 mM IPTG and incubated further at 37 °C for 3 h. Effect of pre-culture incubation time and temperature on ethyl lactate production was analyzed, where the pre-cultures were induced with 0.1 mM IPTG after 3 h incubation at 37 °C and incubated further at 30 °C and 37 °C for 20 h. High-cell density (HCD) culture experiments were conducted by inoculating the aerobically grown pre-cultures in hybrid M9 medium with antibiotics at different optical densities (3.0 as HCD3, 10.0 as HCD10, 20.0 as HCD20, 30.0 as HCD30), varying glucose supplementations (2%, 5%, 10% w/v), different IPTG concentrations (0.01, 0.1, 0.5, 1.0 mM), and temperatures (25 °C, 30 °C, 37 °C); as per experimental design and optimization strategy. HCD cultures were grown under low oxygen condition in triplicates in dual-lock tubes (3 mL culture volumes) which were subjected to ‘intermittent aliquot sampling’ during cultivation. These cultures were externally doped with 2 g/L (D/L) lactate and 10 g/L ethanol, and comparative studies were performed with the undoped HCD cultures for ethyl lactate production. To evaluate the effect of mode of cultivation and to avoid any oxygenation of the fermentation conditions, further experiments were performed with ‘sample-wise cultivation’ of cultures in 2 mL microcentrifuge tubes, where every sample was distinctly cultured in a separate tube, in triplicates.

### Fermentation in a lab-scale bench-top bioreactor

Batch fermentation experiments were conducted in 1 L bench-top bioreactors with working volume of 500 mL (BioFlo/CelliGen 115, New Brunswick Scientific Inc., Enfield, CT, USA). High performing recombinant strains (SSL74 and SSL76) from the strain screening and selection experiments were selected for bioreactor fermentations. Recombinant strains of *E. coli* (SSL74 and SSL76) were inoculated with 100 μL of glycerol master stock culture in a shake flask with 50 ml LB medium containing 100 μg/mL carbenicillin and 20 μg/mL chloramphenicol and incubated for 18 h at 37 °C and 250 rpm. This pre-culture is used to inoculate seed culture for bioreactor fermentations at 2% v/v. Seed cultures of *E. coli* SSL74 and SSL76 were grown in a 1 L bioreactor in LB broth containing 100 μg/mL carbenicillin and 20 μg/mL chloramphenicol. This seed cultures were incubated for 20 h at 30 °C and 200 rpm. To achieve high cell density cultivation, seed cultures were grown aerobically by maintaining dissolved oxygen (DO) level at 40% in a gas flow cascade mode until OD_600_ value reached about 2.7 to 2.9. The resultant cell pellet obtained after centrifugation (Thermofischer Scientific Sorvall Legend XTR Centrifuge) (3973 rpm, 10 min, 4 °C) of the seed culture was washed and suspended in hybrid M9 medium containing 100 μg/mL carbenicillin and 20 μg/mL chloramphenicol. Hybrid M9 medium recipe is provided in supplementary materials. 0.1 mM of IPTG was added after 3 h to a bioreactor with working volume of 500 mL containing hybrid M9 medium, inoculated with high cell density culture (initial OD_600_ was ~ 4 to 5). The pH was maintained at appropriate value by auto-addition of 12 N NaOH and 1 N H_2_SO_4_. A dual-pulse anaerobic fed-batch fermentation experiment was also conducted in bench-top bioreactors with 500 mL as the initial working volume where an additional glucose of 25 g/L was manually fed to the bioreactor at 6 h and 20 h. The rest of the fermentation conditions were identical to the batch fermentation experiment, unless otherwise specified.

### Substrate and metabolite analysis

Glucose, acetic acid, lactic acid, and succinic acid analyses were performed on an Agilent 1100 HPLC equipped with an Aminex 87H column, a Diode-Array Detection (DAD) and a refractive index detector (RID). Culture broths were collected at appropriate time intervals and centrifuged (Eppendorf Centrifuge 5415 D) at 12,000 rpm and 2 min. The supernatants were collected, appropriately diluted, filtered and injected onto an ion exchange HPLC column (an Aminex 87H column). The mobile phase was 5 mM H_2_SO_4_ at a flow rate of 0.6 mL/min. The column temperature was at 70 °C and the RID detection (for glucose quantification) temperature was at 37 °C and DAD detection (for acids quantification) at 210 nm. Enzyme based D(−)/L( +) lactic acid detection kit (R-biopharm) was used to verify the optical purity of lactate from the engineered strains.

### GC–MS analysis

Ester were quantified using GC–MS analysis. For the analyses, analytes in the culture supernatants and/or whole cultures were extracted using chloroform in a 5:4 (v/v) ratio for 10 min at RT, on a vortex machine, in 2 mL microcentrifuge tubes. The mixture was centrifuged at 10,000 rpm for 2 min and the 1 µL of organic layer was injected into the GC system (7890A) coupled with 5975C inert MSD system from Agilent Technologies. The injection was made in a splitless mode. For GC system, hydrogen was used as a carrier gas with a flow rate of 1 mL/min and analytes were separated on DB-5ms column (30 m × 0.25 mm, 0.25 µm) from Agilent Technologies. The oven temperature was ramped with an initial temperature of 50 °C with a 1 °C/min to 58 °C. Next, a 25 °C/min ramp was deployed to 250 °C. Post-run was at 300 °C for 3 min to avoid any interference from remnants^2^. Analytes were detected using selected ion monitoring (SIM) mode. Dynamic SIM strategy was employed as follows: for ethyl lactate, ions 45.0, 46.0, 74.8, 74.9, 75.0, 75.1 were detected from 8.00 to 15.68 min; for ethyl acetate, ions 43.0, 60.8, 60.9, 61.0, 61.1, 71.0 were detected from 2.50 to 8.00 min. 75.0 and 61.0 were used as characteristic ions for ethyl lactate and ethyl acetate, respectively.

### Data analysis

HCD cultures were cultivated in biological triplicates, whereas the fermentation samples were taken as technical duplicates for metabolite and ester analysis. Mean values and standard deviations were calculated by Microsoft Excel standard functions.* P* values used for determining statistical significance of our results were calculated in Microsoft Excel using Student’s t-test.

## Supplementary Information

Supplementary Information.

## Data Availability

All data generated and analyzed during this study are included in this article and its supplementary information.
