# Supplementary material for: Unravelling the hidden power of esterases for biomanufacturing of short-chain esters
Source: Sci Rep. 2023 Jul 4;13:10766. doi: 10.1038/s41598-023-37542-x (PMC10319855; doi:10.1038/s41598-023-37542-x)
Supplement: Supplementary file 1 — Supplementary Information. [file 41598_2023_37542_MOESM1_ESM.docx]

**Supplementary information**

**SI-1a: Schematic representation of the steps followed for correcting non-nucleotide or non-amino acid inclusions from polynucleotide or polypeptide sequences, respectively, of esterases or acyltransferases from *Brettanomyces bruxellensis* in the online databases. The image was created using Microsoft PowerPoint and Microsoft Excel.**

**
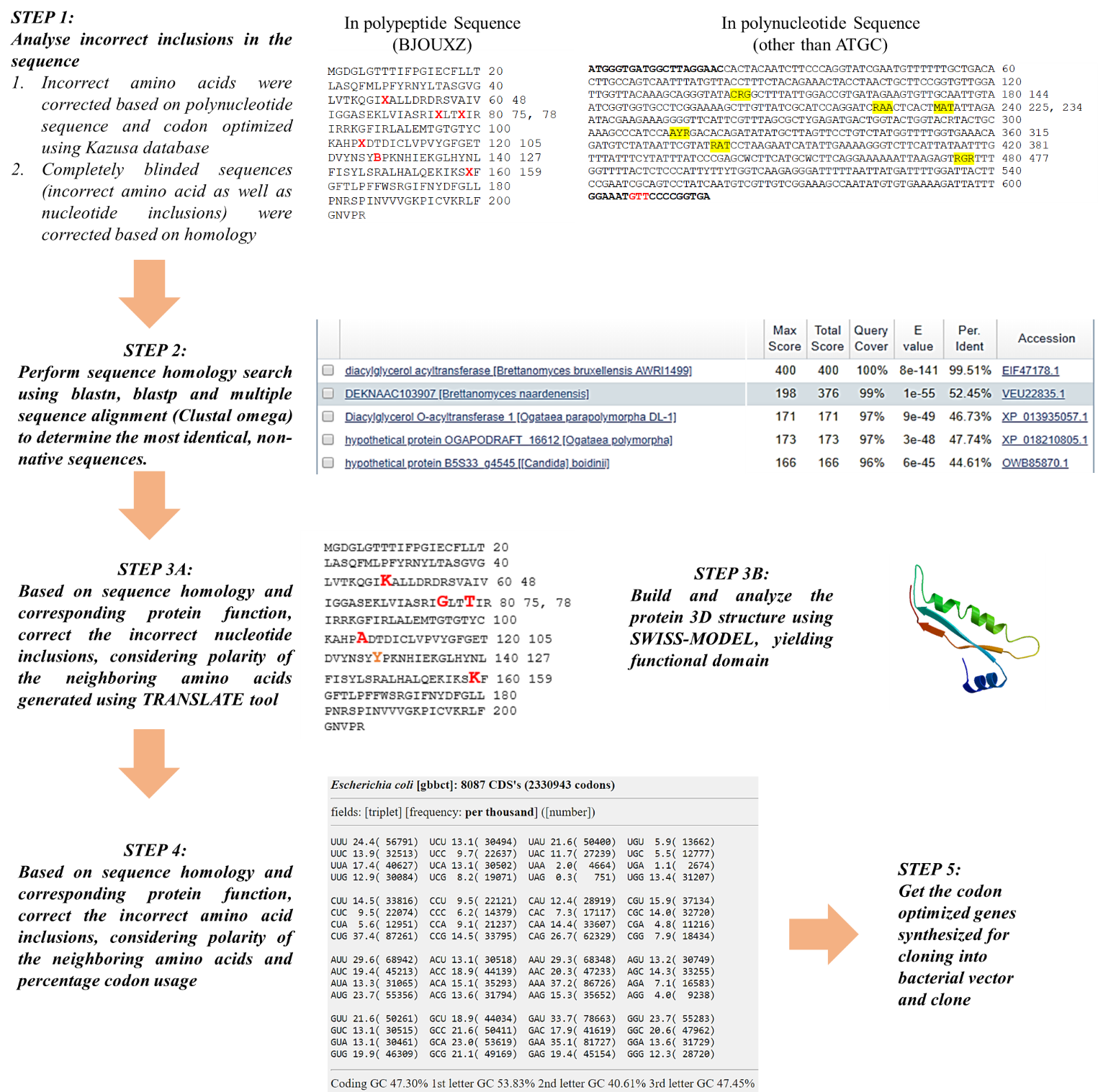
**

**SI-1b: *estA* gene from *Pseudomonas aeruginosa* has native signal peptide. Removal of the signal peptide (*testA*) renders the protein, tEstA more soluble. Comparative SDS-PAGE results of crude cell lysates from recombinant *E. coli* BL21-DE3 strains possessing soluble carbohydrate esterase (CE, positive control), empty plasmid (pET15b, negative control), EstA and tEstA exhibited denser esterase A band with tEstA, indicating increased cytoplasmic soluble fraction over membrane bound EstA, indicating successful protein engineering. The image was created using Microsoft PowerPoint. The gel picture was captured using a trans-illuminator.**

**
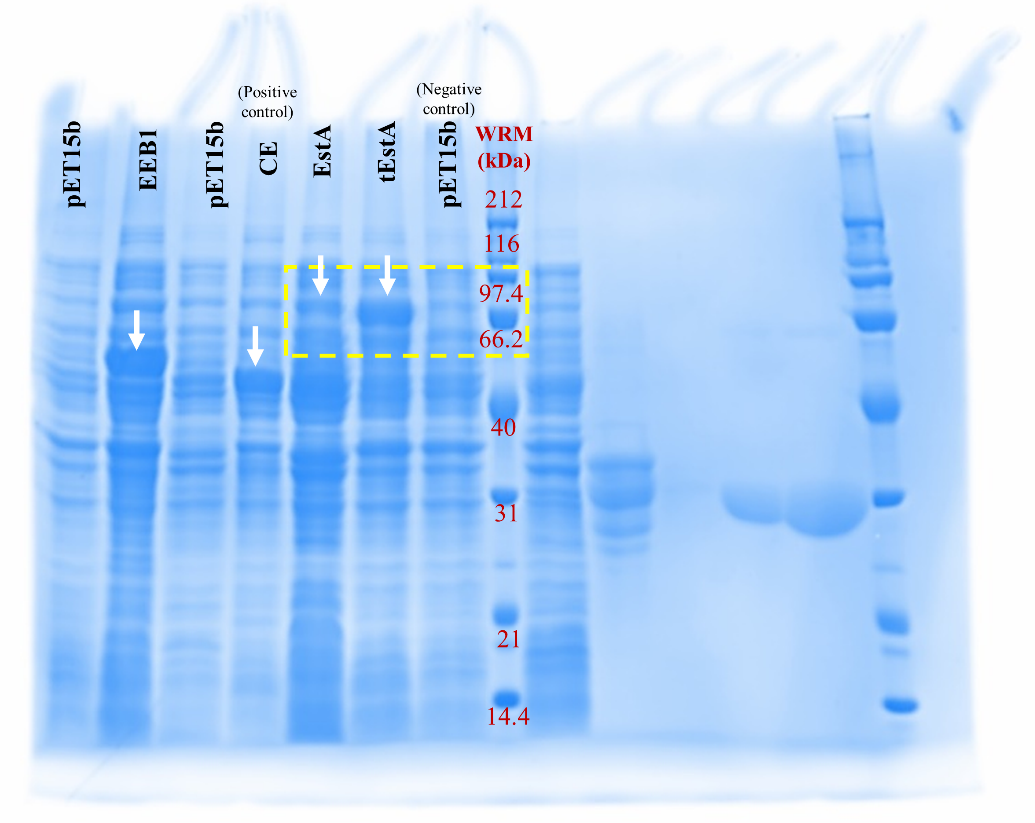
**

**
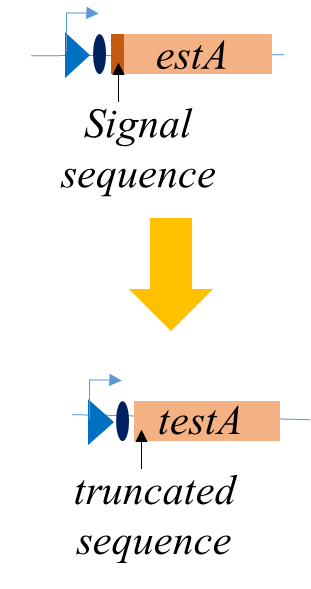
**

**SI-2: Schematic representation of the cloning strategy followed for constructing Phase I and Phase II *E. coli* strains using pET15b as a base vector. The image was created using Microsoft PowerPoint.**

**
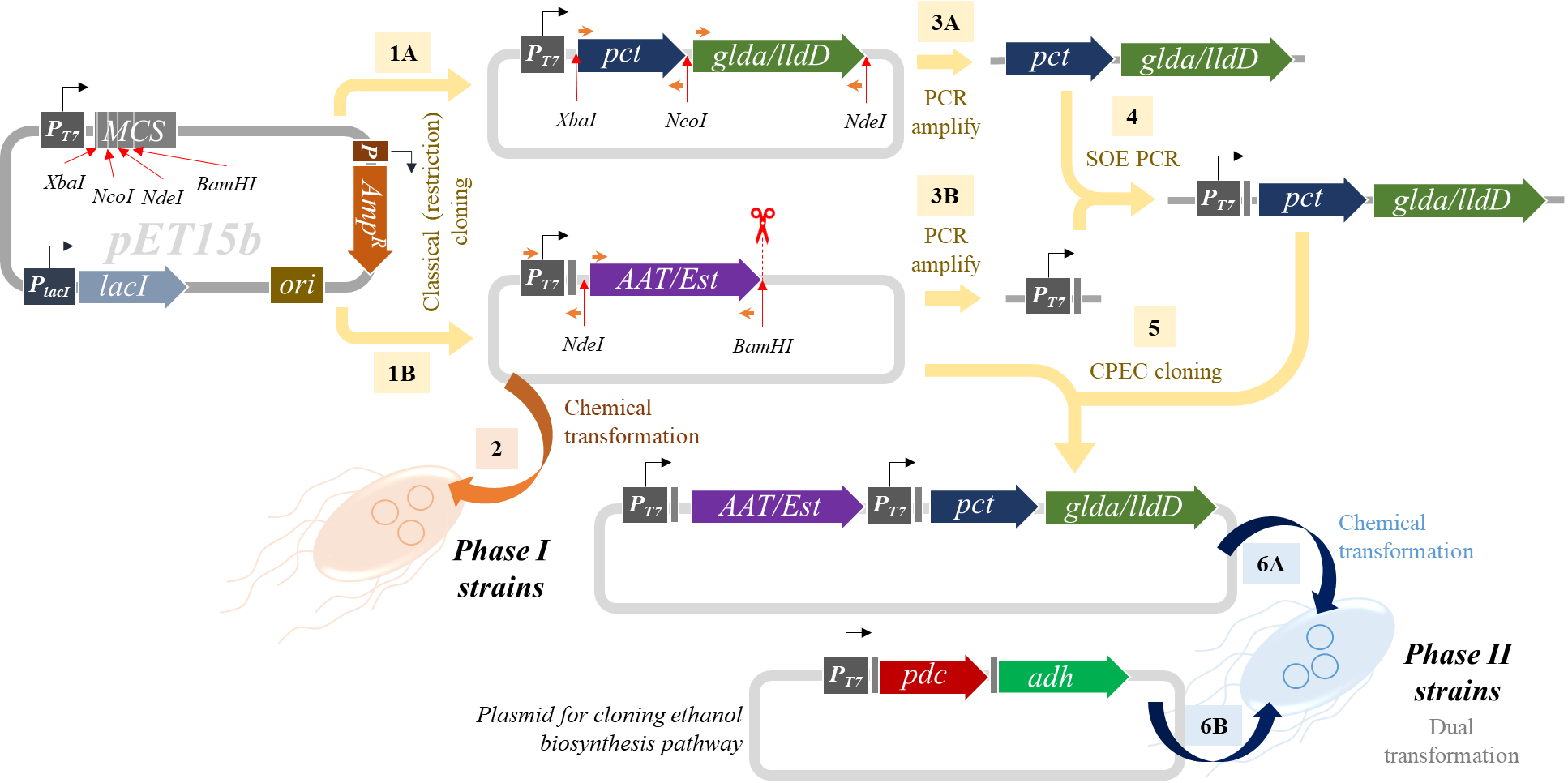
**

**SI-3: a) Lactate and ethanol titers from *E. coli* BL21-DE3 strains transformed with empty pET15b plasmid over 46 h of high cell density cultivation (OD_600_ of 3.0, HCD3), b) Comparative screening of Phase I *E. coli* strains possessing different microbial esterases and acyltransferases with HCD3 cultivation and aliquote sampling strategy for ethyl lactate production. The image was created using Microsoft PowerPoint and Microsoft Excel.**

**
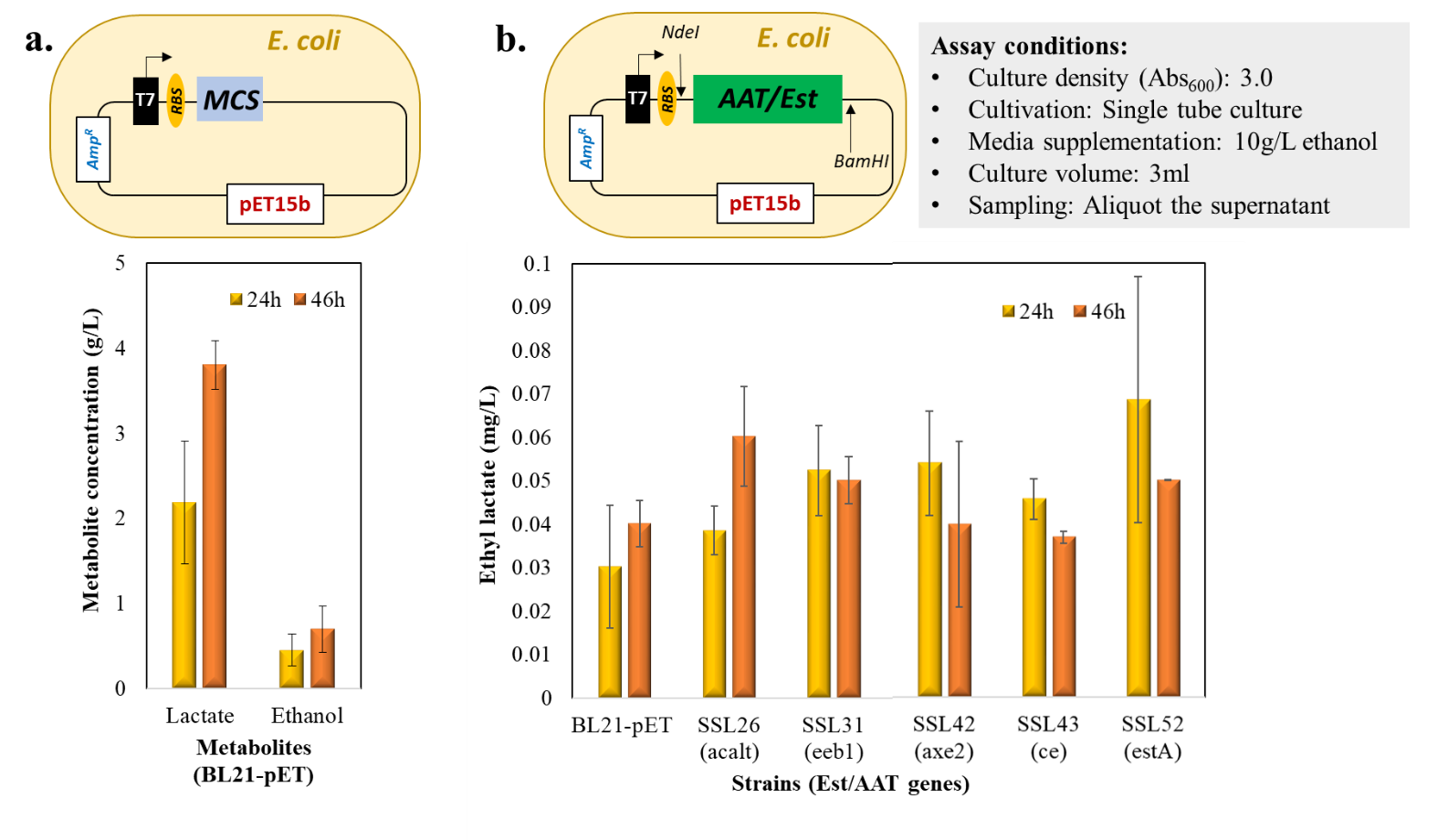
**

**SI-4: Comparative ester and precursor production analyses of HCD3 (a) and HCD10 (b) cultivated Phase II strains after 24h incubation. The image was created using Microsoft PowerPoint and Microsoft Excel.**


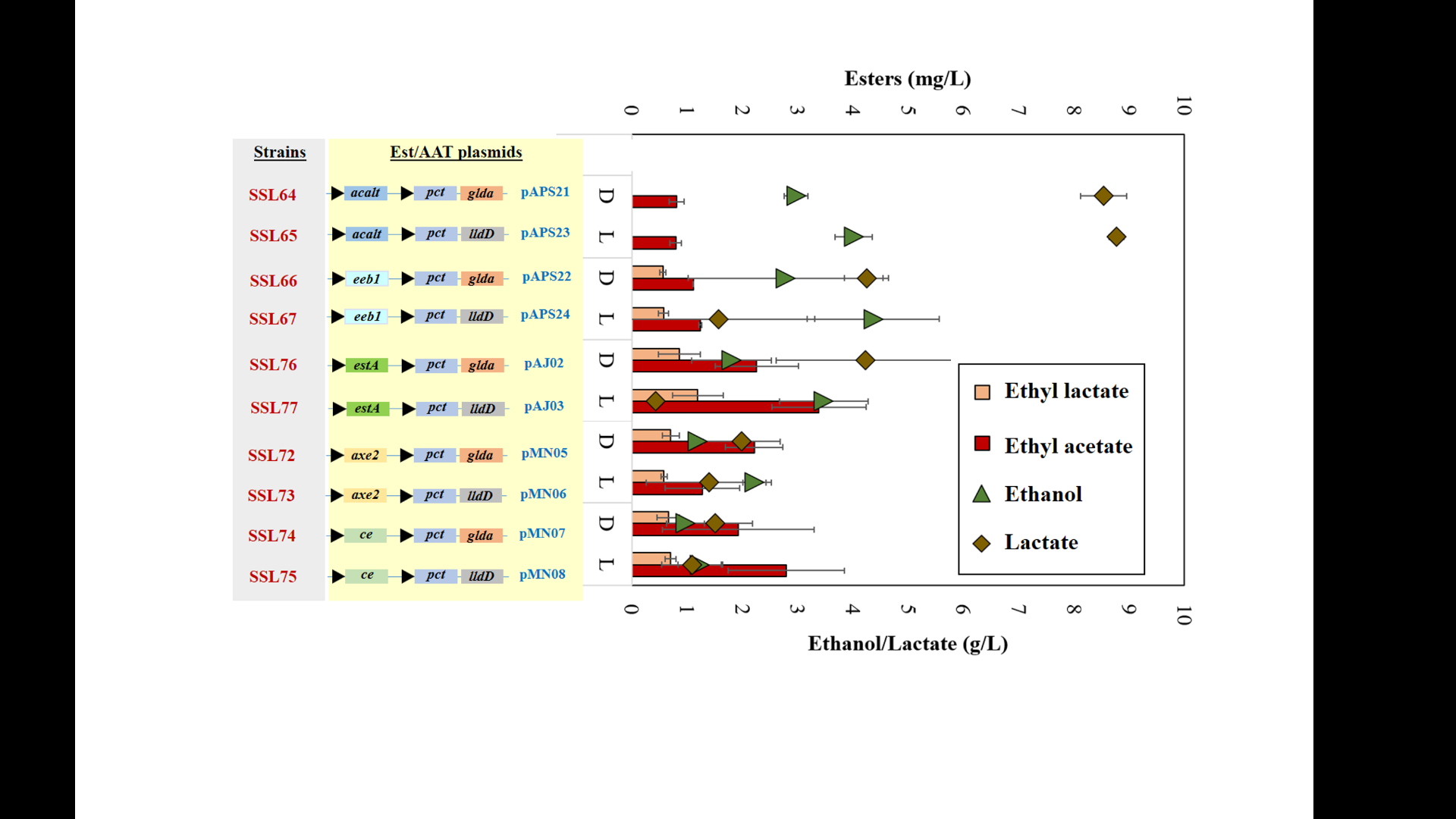
**a)**

**b)** HCD10 was conducted with and without external supplementation of ethanol and corresponding D- or L-lactic acid. The results showed that the doped samples produced almost double the amount of ethyl lactate than undoped ones, attributing to the ample availability of the precursors for esterification.

**
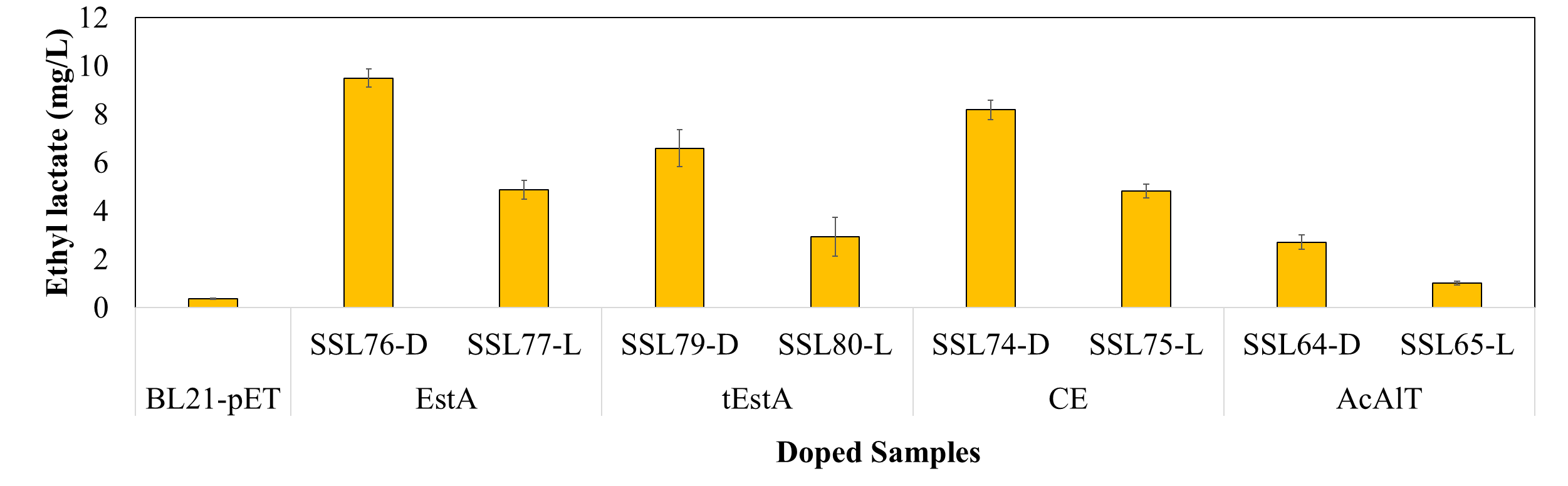
**

**SI-5: HPLC analysis of SSL76 and SSL77 strains cultivated with HCD10 for corresponding lactate (a) and ethanol (b) production over 72h. The image was created using Microsoft PowerPoint and Microsoft Excel.**

**
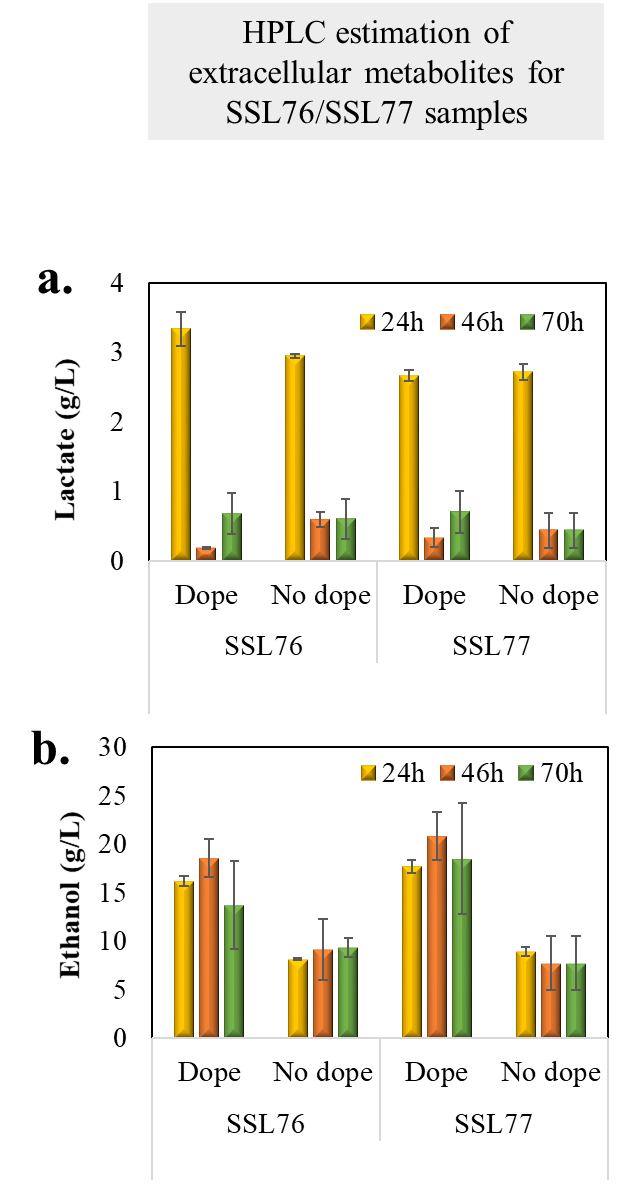
**

**SI-6: Optimization of fermentation conditions: (a) SSL76 strain was subjected to different cell densities (OD_600_ of 3.0 (HCD3), 10.0 (HCD10), 20.0 (HCD20), 30.0 (HCD30)) to estimate optimum HCD for higher ester production. HCD10 was also analyzed for improved expression with different IPTG concentrations where 0.1mM IPTG was found to be the most optimum. (b) Varying concentrations of glucose were tested for increasing ethyl lactate concentrations with HCD10. Small increments were observed with increasing cell densities to HCD20 and HCD30, but the increment was not proportional to the increments in cell densities. (c) Effect of pre-culture incubation temperatures on ethyl lactate production was analyzed over the period of 72h, which showed significantly higher production at 30℃. (d) Culture supernatant as well as the biomass were tested for ethyl lactate concentrations. 20-23% ethyl lactate was observed to be retained within the cell biomass whereas rest is released into the spent medium (culture supernatant). (e) Effect of HCD culture incubation temperature was analyzed on ethyl lactate production, where the highest production was observed with 37℃ where the pre-culture was incubated at 30℃, as optimized. The image was created using Microsoft PowerPoint and Microsoft Excel.**

**
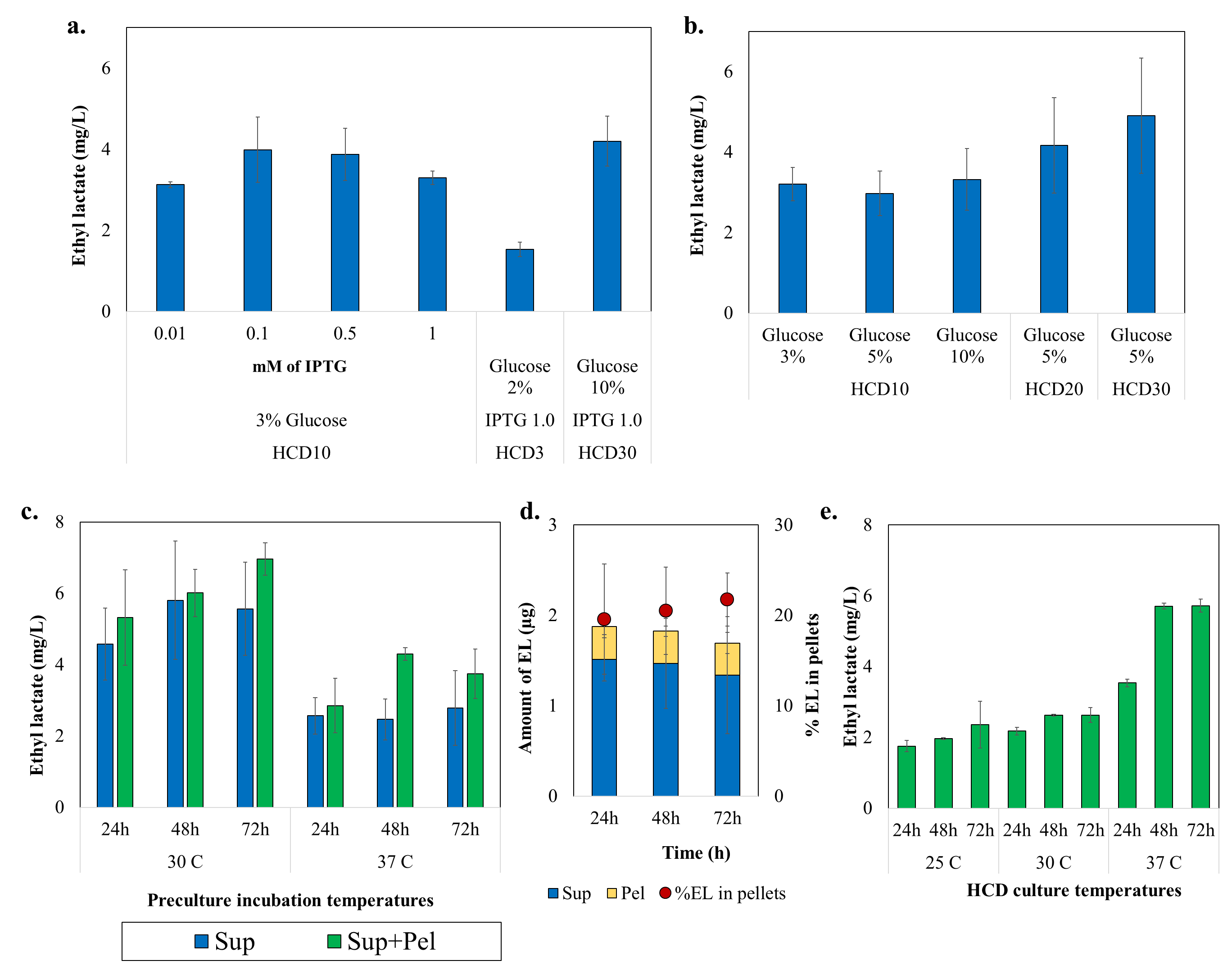
**

**SI-7: List of primers used**

| **Primers** | **Sequences** | **Amplicon binding (purpose)** |
| --- | --- | --- |
| P1 | atatagctacaaaaccggtatctaagcgaaattaatacgactcactatagg | T7 promoter forward (for CPEC) |
| P2 | gctgccgcgcggcaccaggc | T7 terminator reverse |
| P3 | gtaattatgggtactttcctcatgctgccgcgcggcaccag | T7 terminator reverse (for CPEC) |
| P7 | atttcatatgatgccgagctggggtttcc | *acalt* gene forward (NdeI cloning) |
| P8 | attcggatccttagataccggttttgtag | *acalt* gene reverse (BamHI cloning) |
| P9 | gattcatatgatgttccgtagcggttac | *eeb1* gene forward (NdeI cloning) |
| P10 | atttggatccttacagaaccagctcatc | *eeb1* gene reverse (BamHI cloning) |
| P11 | tcttctagatttgtttaactttaagaaggagaatgaggaaagtacccataat | *pct* gene forward (XbaI cloning) |
| P12 | ttcccatggtctccttcttaaagttaaacaaattagcttttcatctctttcag | *pct* gene reverse (NcoI cloning) |
| P13 | attacatatgtttgtttaactttaagaaggagaatgataaggatggctttaaa | *estA* gene forward (NdeI cloning) |
| P14 | aatcggatccttagaaatccaaggacagagc | *estA* gene reverse (BamHI cloning) |
| P15 | attaccatggatgaccaagatcatcacctc | *gldA101* gene forward (NcoI cloning) |
| P16 | aatacatatgttaagcccacttttccttgtag | *gldA101* gene reverse (NdeI cloning) |
| P17 | attaccatggatgattatttccgcagccag | *lldD* gene forward (NcoI cloning) |
| P18 | tttacatatgctatgccgcattccctttcg | *lldD* gene reverse (NdeI cloning) |
| P23 | tttacatatgatggatcaatatcca | *axe2* gene forward (NdeI cloning) |
| P24 | atttggatccttaaattccggcgacatag | *axe2* gene reverse (BamHI cloning) |
| P25 | atttcatatgatggagtctgttaagccac | *ce* gene forward (NdeI cloning) |
| P26 | actaggatcctcagaagctttgttcgatgcc | *ce* gene reverse (BamHI cloning) |

**SI-8: Effect of pH on *in vitro* enzymatic esterification. Phosphate buffers with varying pH (6, 7, 7.6, 8) were compared for the production of ethyl lactate, from ethanol and lactic acid (10 g/L each) *in vitro*, using a crude cell lysate of induced SSL79 as a source of tEstA, at 37 ℃. Results exhibited the highest ethyl lactate titers at pH 6.0. The image was created using Microsoft Excel.**

**
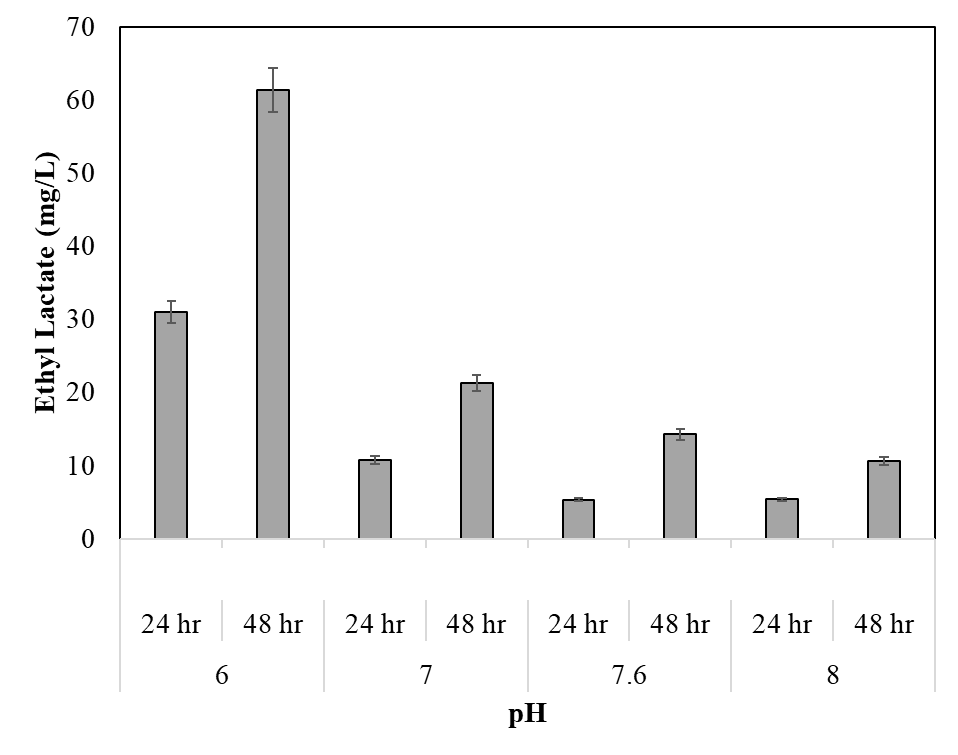
**
